# Biological and Phytochemical Investigations on* Caesalpinia benthamiana*, a Plant Traditionally Used as Antimalarial in Guinea

**DOI:** 10.1155/2017/9438607

**Published:** 2017-09-10

**Authors:** Jean Loua, Mohamed Sahar Traore, Aissata Camara, Mamadou Aliou Balde, Louis Maes, Luc Pieters, Aliou Mamadou Balde

**Affiliations:** ^1^Research and Valorization Center on Medicinal Plants, Dubreka, Guinea; ^2^Department of Pharmacy, University Gamal Abdel Nasser of Conakry, Conakry, Guinea; ^3^Laboratory of Microbiology, Parasitology and Hygiene (LMPH), Faculty of Pharmaceutical, Biomedical and Veterinary Sciences, University of Antwerp, Universiteitsplein 1, 2610 Antwerp, Belgium; ^4^Natural Products & Food Research and Analysis (NatuRA), Department of Pharmaceutical Sciences, University of Antwerp, Universiteitsplein 1, 2610 Antwerp, Belgium

## Abstract

*Caesalpinia benthamiana* is widely used as antimalarial in Guinean traditional medicine. Leaf extracts of the plant were tested for their in vitro antiprotozoal activity against* Trypanosoma brucei brucei and T. cruzi *and the chloroquine-sensitive Ghana strain of* Plasmodium falciparum *along with their cytotoxicity on MRC-5 cells. The methanolic extract showed the strongest antiprotozoal activity against* P. falciparum *(IC_50_ 4 *μ*g/ml), a good activity against* T. brucei* (IC_50_ 13 *μ*g/ml), and a moderate activity against* T. cruzi* (IC_50_ 31 *μ*g/ml) along with an IC_50_ on human MRC-5 cells of 32 *μ*g/ml. Bioassay-guided fractionation from the methanolic extract led to antiplasmodially active subfractions. A prospective, placebo-controlled ethnotherapeutic trial assessed the antimalarial effectiveness and tolerability of* C. benthamiana* syrup administered orally to children with uncomplicated malaria as compared with chloroquine syrup. Phytochemical screening of the leaf extracts indicated the presence of flavonoids, terpenoids, tannins, saponins, and iridoids.

## 1. Introduction

Malaria is the most important parasitic disease of human beings. It is transmitted in more than 100 countries inhabited by roughly 3 billion people [1]. Approximately 3.2 billion people, almost half the world's population, are exposed to malaria risk [2]. According to the most recent statistics of the World Health Organization's World Malaria Report 2015, an estimated 214 million new cases of malaria and 438,000 deaths had been recorded worldwide [2]. More than 85% of malaria cases and 90% of malaria deaths occur in Sub-Saharan Africa, mainly in young children (i.e., those younger than 5 years old) [1].

A dramatic revival of malaria is ongoing due to the increasing resistance of vectors to insecticides and to the progressive resistance of the parasite, mainly* Plasmodium falciparum*, to drugs [3, 4]. Moreover, resistance to artemisinins has now been detected in five countries in the Greater Mekong subregion: Cambodia, Laos, Myanmar, Thailand, and Vietnam [2].

Guinea is highly endemic for malaria, with a prevalence in children younger than 5 years old of 44% in a 2012; Malaria is the main cause of visits to health facilities, accounting for more than 30% of visits to public health facilities [5]. Due to financial, geographical, and/or cultural obstacles, most Guinean people especially in rural areas are still not accessing the services they need to prevent and treat malaria and affected populations turn towards traditional medicine and medicinal plants to solve their health problems.

In the search for new therapeutic agents against malaria from the Guinean flora, a series of ethnobotanical investigations were realized by the Guinean “Centre de Recherche et de Valorisation des Plantes Médicinales (CRVPM) de Dubréka.” Among the collected plants* Caesalpinia benthamiana *(Baill.) Herend. & Zarucchi (Synonym:* Mezoneuron benthamianum *Baill.) (Fabaceae) was selected for an ethnopharmacological investigation.

*C. benthamiana *is well-known in Guinean traditional medicine for the treatment of various illness including malaria [6] and preliminary biological studies showed a remarkable antiplasmodial activity of this plant species with an IC_50_ value of 5.8 *μ*g/ml against* Plasmodium falciparum *[7].

The aim of this study was to determine the in vitro antiprotozoal activity of* C. benthamiana* extracts (Table 1), to carry out a preliminary phytochemical investigation of the leaves of* C. benthamiana*, and to conduct a prospective, placebo-controlled ethnotherapeutic trial to assess the antimalarial effectiveness and tolerability of a* C. benthamiana* syrup administered orally to children with uncomplicated malaria as compared to chloroquine syrup.

## 2. Material and Methods

### 2.1. Plant Material

The leafed twigs of* C. benthamiana *were harvested in the prefecture of Dubréka in September 2006. The voucher specimen was authenticated by the Department of Botany of the “Centre de Recherche et de Valorisation des Plantes Médicinales (CRVPM), Dubréka” and deposited in the herbarium of this Center (No. 27HK411). The sample was dried at room temperature and pulverized.

### 2.2. Ethnotherapeutic Evaluation

The ethnotherapeutic evaluation was made mainly on the basis of the knowledge and recommendations of the traditional healer owning the recipe and within his community where* C. benthamiana* is largely used either for the treatment of malaria and other infectious diseases.

### 2.3. Study Site and Participants

This ethnotherapeutical trial took place at Dubréka, an area of high malaria transmission in Guinea. The study was conducted between June and July 2007, within the district hospital of Dubréka.

### 2.4. Entry Criteria

Subjects with a positive thick drop test were included if they fulfilled all the following inclusion criteria:

1. Age between 5 and 15 years old,
2. Mono-infection with* P. falciparum* infection,
3. Parasitaemia ≥ 1000 asexual parasites/*μ*l,
4. Haemoglobin (Hb) ≥ 5 g/dl,
5. Availability during the study,
6. Fever ≥ 37.5°C (or history of fever during the past 24 h).

Subjects were excluded if they had any of the following:

1. A history of allergy to the study drugs,
2. Having taken an antimalarial drug in the previous 72 h,
3. Fever due to diseases other than malaria or other known underlying chronic diseases,
4. Severe illness,
5. Medications that may interfere with antimalarial drugs,
6. Presenting any dangerous signs of severe malaria [8].

All parents/guardians were requested to give written consent before entering the study. The aims and procedures of the study were explained to each patient or parent/guardian and his/her understanding was confirmed by interview before written or, in case of illiteracy, thumb-printed consent in the presence of an unbiased witness. This study was approved by the Ethical Committee of the Center.

### 2.5. Clinical Monitoring

Clinical examination was directed by the research team doctor and targeted the body weight, temperature, palpation, and auscultation.

### 2.6. Laboratory Procedures

At enrolment, 5 ml of venous blood was drawn to confirm the diagnosis of* P. falciparum *malaria with Giemsa stained thick and thin smears, stored blood on filter paper for further parasite genotyping, and to obtain a complete hemogram (manually), haematocrit (%), haemoglobin concentration (g/dl), and blood glucose (mmol/l). The smears and filter paper blood samples were also collected from finger pricks on days 0, 3, 7, 14, and 28. The smears were read by a laboratory technician experienced in malaria diagnosis. Parasitaemia was calculated based on the number of asexual forms observed in 200 leucocytes and then multiplied by 40.

### 2.7. Galenic Formulation

Based on the traditional way of using* C. benthamiana*, 3000 g of the leaf powder was extracted by percolating with 2 × 5 l of ethanol 70° during 48 h. The resulting extracts were concentrated under reduced pressure at 40°C to yield 220 g of the wimpy crude extract which was mixed with 500 ml of hot water, and then a sufficient quantity of simple syrup was added to get 3330 ml of Caesalpinia syrup.

The chloroquine syrup was purchased at the Central Pharmacy of Guinea (PCG).

### 2.8. Treatment Assignment

In order to compare the two regimens, children were divided into two groups assigned to receive either syrup of* C. benthamiana* for group I or chloroquine syrup (5 mg/ml) for group II. Group I received 2 × 15 ml (morning and evening) per day of* C. benthamiana* syrup orally from day 1 (D1) to day 5 (D5) (150 ml). The second group received 10 mg/kg of chloroquine at D1 and D2, then 5 mg/kg at D3 [9]. Children were observed for 30 min after each drug administration. Treatments were readministered if the child vomited.

*Follow-Up*. Subjects were followed up on days 0, 3, 7, 14, and 28 for clinical and laboratory assessments described above.

### 2.9. Phytochemical Investigation

A portion of the leaf powder was subjected to phytochemical analysis, using standard chemical tests. Phytochemical screening of the crude extract was carried out employing standard methods and tests [10].

For the preliminary phytochemical analysis, 100 g of powdered leaves of* C. benthamiana* was macerated with 500 ml of EtOH 70° (48 h). The extract was concentrated under vacuum and dried. The dried extract was tested for the presence of different phytoconstituents, namely, terpenoids, alkaloids, coumarins, tannins, anthraquinones, flavonoids, anthocyanins, saponins, and iridoids, by TLC using precoated silica gel plates (Merck) and common coloration and precipitation reactions [11, 12].

### 2.10. Fractionation

The active extract and its fractions were consecutively submitted to a bioassay-guided fractionation by column chromatography on silica gel 60–200 mesh (Merck) with dichloromethane/ethyl acetate as eluent, with a gradient of increasing polarity. Analytical TLC was performed on precoated silica gel 60 F_254_ plates (Merck; 0.25 mm) with the following mobile phases: dichloromethane/toluene 7 : 3 and dichloromethane 100%.

### 2.11. Preparation of the Extracts

Powdered leafed twigs of* C. benthamiana* (50 g) were macerated with 500 mL of either methanol, distillated water, or CH_2_Cl_2_ for 72 h to give three extracts which were evaporated to dryness in vacuo at 40°C to give Mb1 (12.46 g); Mb2 (6.68 g); Mb3 (5.15 g), respectively.

### 2.12. In Vitro Antiprotozoal Screening

The extracts were tested for their antiprotozoal activity and cytotoxicity (MRC-5 cells) according to a previously described method [13]. The antiplasmodial activity of extracts and fractions was evaluated in vitro against a chloroquine-sensitive strain of* Plasmodium falciparum* (3D7) using a modified procedure according to Makler et al. [14] (lactate dehydrogenase assay).

Positive controls were tamoxifen and suramin (Sigma-Aldrich) for MRC-5 and* T. brucei*, respectively, and benznidazole and chloroquine (WHO-TDR, Geneva, Switzerland) for* T. cruzi* and* P. falciparum*, respectively. [7].

### 2.13. Bioassay-Guided Fractionation

The active extract and fractions were consecutively submitted to bioassay-guided fractionation by column chromatography on silica gel 60–200 mesh (Merck) as indicated above. The fractions were tested against* P. falciparum* and* T. cruzi*.

### 2.14. Statistical Analysis

Statistical analyses were performed using Excel, Mann–Whitney test, and SPSS version 20.0.

Significance was set at *p* < 0.05.

## 3. Results

### 3.1. Biological Activity

#### 3.1.1. In Vitro Antiprotozoal Activity

The most active antiprotozoal extract was the methanolic extract (Mb1), which showed a pronounced activity against* P. falciparum *(IC_50_ 4 *μ*g/ml), a moderate activity against* T. brucei* (IC_50_ 13 *μ*g/ml), and a weak activity against* T. cruzi* (IC_50_ 31 *μ*g/ml); it was cytotoxic on human MRC-5 cells with an IC_50_ of 32 *μ*g/ml. The apolar extract Mb3 was four time less active against* P. falciparum* than Mb1 while Mb2 was inactive (IC_50_ > 64 *μ*g/ml). Indeed, Mb2 was only active against* T. brucei* and less cytotoxic (IC_50_ > 64 *μ*g/ml) than Mb1 and Mb3 (IC_50_ 32 and 33 *μ*g/ml, resp.).

A bioassay-guided fractionation led to 7 subfractions: Mb1.1 to Mb1.7. The subfractions Mb1.1 and Mb1.2 were the most antiplasmodially active with an IC_50_ of 2 *μ*g/ml, each, along with a cytotoxicity IC_50_ of 29 and >64 *μ*g/ml, respectively. A combination of these two subfractions (Mb1.1m) was refractionated yielding 8 subfractions, Mb1.1m1 to Mb1.1m8. All of these were less active than Mb1.1 or Mb1.2. However, a strong antiplasmodial effect was observed for Mb1.1m4 and Mb1.1m5 (IC_50_ 4.24 and 4.00 *μ*g/ml, resp.), while a good activity was recorded for Mb1.1m3 and Mb1.1m2 (IC_50_ 6.49 and 7.18 *μ*g/ml, resp.). The other subfractions were moderately active (IC_50_ 17.42 to 19.85 *μ*g/ml).

On the other hand, Mb1.1, Mb1.2, and Mb1.3 showed the best antitrypanosomal activity against* T. cruzi* (IC_50_ 8, 16, and 10 *μ*g/ml, resp.) and* T. brucei* (IC_50_ 32, 35, and 33 *μ*g/ml, resp.). However, with an IC_50_ of 29 *μ*g/ml, Mb1.1 was also the most cytotoxic fraction among all the tested subfractions (Figure 2).

### 3.2. Ethnotherapeutic Evaluation

#### 3.2.1. Baseline Characteristics

**Figure 1:**
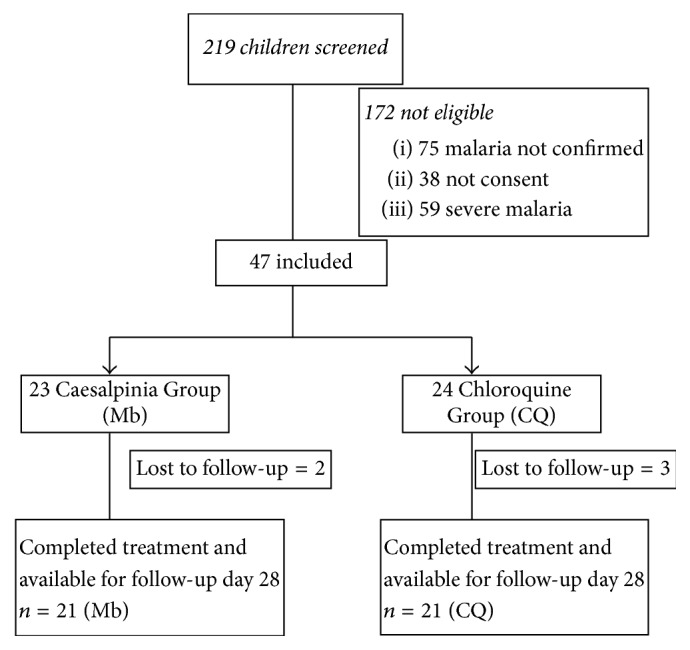
Trial profile

A total of 219 children were screened and from these 42 patients were enrolled in the study with 21 receiving chloroquine (CQ group) and 21 others receiving the* C. benthamiana *syrup (Mb group) (Figure 1). The mean parasitaemia of the patients was not significantly different (*p* 0.82): 1847,76 ± 1629,38 for the Mb group (group I) and 1753,95 ± 1557,99 for the CQ Group (group II). The patient demography and baseline characteristics are summarized in Table 2. It is important to note that most of the patients had consumed a paracetamol-based antipyretic before their enrolment.

#### 3.2.2. Efficacy

A significant parasitic load reduction was noticed from day 0 to day 28 for both Mb and CQ treatments (Table 3; Figure 3). No significant difference was observed between the two treatment groups on follow-up days 3, 7, 14, and 28 with *p* values of 0.96; 0.77; 0.72; and 0.79. However, parasite clearance was observed for one patient at D7, two patients at D14, and four patients for D28 in the* C. benthamiana* arm and only four patients at D28 in the chloroquine arm.

#### 3.2.3. Tolerance

During the 28-day follow-up period, both treatments with Mb and CQ were well tolerated with the majority of adverse effects of mild or moderate severity, and consistent with symptoms attributable to malaria. The frequency of individual adverse events was generally similar between the two treatments, although the incidence of posttreatment adverse events (AEs) was slightly higher in patients treated with CQ (Table 3). Among the laboratory parameters, the concentration of mean Hb fell from 8.65 g/dl (Mb) and 9.43 g/dl (CQ) on day 0 to 7.78 g/dl (Mb) and 8.17 g/dl (CQ) on day 7, before increasing progressively thereafter.

The haematocrit values decreased from D0 to D14 before increasing at D28. The blood glucose value did not change significantly in both treatments.

### 3.3. Phytochemical Screening

A qualitative investigation on the leaf extract of* C. benthamiana* revealed the presence of flavonoids, terpenoids, tannins, saponins, and iridoids. Based on the intensity of their coloration or precipitation during the screening, it can be assumed that tannins and flavonoids are among the majority constituents of* C. benthamiana*.

## 4. Discussion

Billions of people in developing countries still use traditional medicinal plants to fulfill their primary health care needs [6, 15, 16]. In the treatment of malaria, the traditional medicines have been used for thousands of years and have been leading sources of important antimalarial drugs known today [6, 17, 18].

### 4.1. In Vitro Antiprotozoal Screening

Of the three tested extracts, the aqueous one was less active (IC_50_ > 64 *μ*g/ml) against* P. falciparum*. The methanolic extract showed the highest antiplasmodial activity (IC_50_ 4 *μ*g/ml) which was in accordance with that of the methanolic extract (IC_50_ 5.84 *μ*g/ml) previously described [7]. Paradoxically, the aqueous extract is the only one widely and well used in Guinean traditional medicine, suggesting that the active compounds could be less hydrosoluble. This is in agreement with a recent report on the moderate activity of the hydroethanolic extract (IC_50_ 22.5–32.6 *μ*g/ml, depending on the batch) and the pronounced activity of the resulting precipitate (IC_50_ 6.5 *μ*g/ml) [8].

Upon consideration of these results, it could be assumed that the antiplasmodial constituents could be a mixture of polar and apolar active compounds. Due to the fact that the methanolic extract was 4 time more active than the dichloromethane extract, along with the decreasing activity observed during the bioassay-guided fractionation of the 2 active subfractions (Mb1.1 and Mb1.2.), the antiplasmodial properties of the leaf extract were most likely related to the presence of semipolar constituents which probably act synergistically.

### 4.2. Phytochemical Investigations

The phytochemical analysis of the 70% EtOH leaf extract of* C. benthamiana *revealed the presence of flavonoids, terpenoids, tannins, saponins, which was in accordance with previous reports [18–22], and iridoids. Since the presence of iridoids in the Fabaceae family is not so usual, further studies are essential to find out their presence in* C. benthamiana. *However, iridoid glucosides such as javanicosides A and B have been described in the leaves and stem bark of another Fabaceae plant species,* Parkia javanica *[23]. Based on the intensity of coloration or precipitation, the leaf extract may be rich in tannins and flavonoids. The absence of anthraquinones in our samples contrasted with described previous report by [22]; this is probably due to the different geographical origin of the tested samples.

The presence of mezobenthamic acids A and B and neocaesalpin H (terpenoids), kaempferol and quercetin (flavonoids), resveratrol, gallic acid, and its ethyl ester, *β*-sitosterol glucoside, and 13*β*-hydroxypheophorbide has recently been described in* C. benthamiana *[8].

Since the leaf extract contains many different classes of compounds, the antimalarial effect of the extracts could be related to the combined effect of both polar and apolar constituents. Although the tannin-rich fractions of* Punica granatum* did not inhibit the growth of* P. falciparum*, their presence could enhance the solubility of hydrophobic molecules and drugs [24].

On the other hand, triterpenoid and steroid saponins have been found to be active against several infectious protozoa such as* P. falciparum *[25].

**Figure 2:**
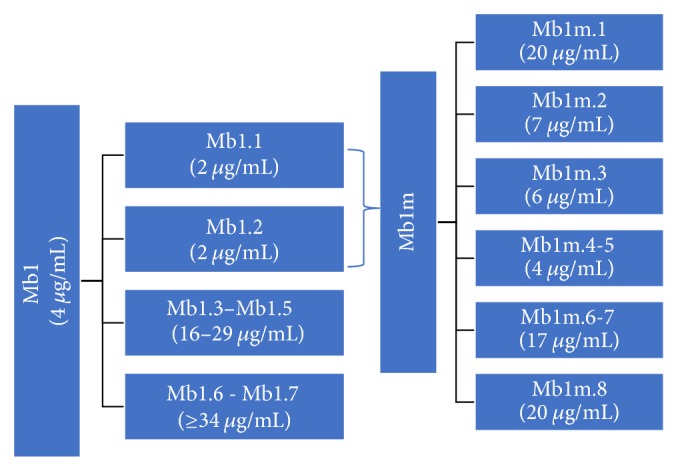
Bioassay-guided fractionation: In vitro antimalarial activity of fractions and subfractions of* C. benthamiana *(*M. benthamianum*, Mb).

Based on the fact that the MeOH extract was more active in vitro than aqueous or CH_2_Cl_2_ extracts, and in order to find the active principles, a bioassay-guided fractionation was performed, which led to two subfractions with a significant activity (IC_50_ 2 *μ*g/ml for each). But when a second fractionation was done from the two-combined active subfractions, the antimalarial effect observed for each of the active fractions decreased as compared with the mother subfraction (Figure 2). This decreasing activity during successive fractionations could be in favor of a synergistic effect of different active constituents. This is supported by the fact that* C. benthamiana* was found to contain gallic acid derivatives which have been described to exert a significant antiplasmodial effect [18, 26–29]. Recently, it was found that the antimalarial compounds belong to several phytochemical classes including flavonoids, pheophorbide and gallic acid derivatives, contributing to the global antiplasmodial activity of the hydroalcoholic extract against* P. falciparum* [8].

### 4.3. Therapeutical Evaluation

A weak clearance of parasitaemia was evident for both treatments, and the progressive and significant reduction exerted by the* C. benthamiana* preparation on the parasitaemia burden supports the traditional use of the plant in the management of malaria.

**Figure 3:**
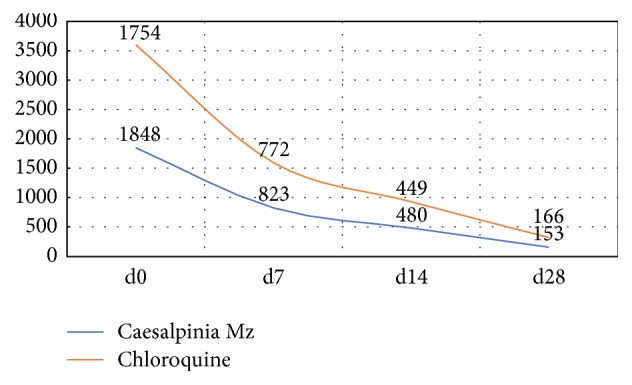
Evolution of parasitaemia during the treatment.

The treatment with the Caesalpinia (Mezoneuron) syrup was safe and able to reduce the parasitaemia burden in children of 5–15 years.* C. benthamiana* appeared to be as efficient as CQ for the treatment of uncomplicated* P. falciparum* malaria (Figure 3). However, the noninferiority of the Mezoneuron syrup as compared with the chloroquine syrup could be related to the chloroquine-resistance recorded in Guinea since 2001 [30].

In addition to the recent publication on this plant species, the therapeutic and antiprotozoal evaluations along with the bioassay-guided fractionation support the traditional use of the leaf of* C. benthamiana* as antimalarial. Since the recent isolation and identification of 13*β*-hydroxypheophorbide as the most active compound in the crude extract of the plant among others such as the major compounds ethyl gallate and quercetin [8], the standardization of a phytomedicine is thus facilitated.

Aiming to valorize this plant species, all the above results could help to improve the antiplasmodial activity as well as the traditional pharmaceutical form of* C. benthamiana *into a standardized phytomedicine which will be submitted to clinical trials in Guinea.

## Figures and Tables

**Table 1:** In vitro antiprotozoal and cytotoxic activity of *Caesalpinia benthamiana* fractions (Mb).

Antiprotozoal activity, IC_50_ (*μ*g/ml)				MRC-5 IC_50_ (*μ*g/ml)
Fractions	*P. falciparum *	*T. brucei *	*T. brucei *	
Mb1	4	31	13	32
Mb2	>64	>64	32	>64
Mb3	16	34	>64	33
Chloroquine	0.047 *μ*M			
Suramin			0.035 *μ*M	
Benznidazole		2.0 *μ*M		

**Table 2:** Baseline characteristics of enrolled patients on day 0.

Patient characteristics	Group I: Mz (*n* = 21)	Group II: CQ (*n* = 21)
Gender: male/female (ratio)	10/11 (0.91)	12/9 (1.33)
Age groups (%)		
5-6	2 (9.52%)	2 (9.52%)
7-8	1 (4.76%)	4 (19.05%)
9-10	6 (28.57%)	4 (19.05%)
11-12	5 (23.81%)	8 (38.09%)
13–15	7 (33.33%)	3 (14.29%)
Mean of body temperature, °C (maxima-minima)	36.87 (37.7–36.2)	37.01 (37.5–36.0)
Geometric mean Parasitaemia/mm^3^ (±SD)	1847,76 (1629,38)	1753,95 (1557,99)
Haemoglobin concentration, mean (g/dl)	8.65 (12–6)	9.43 (14–5)
Hematocrit (%)	35.48 (42–26)	33.86 (40–26)
Blood glucose (mmol/l)	5.89 (7.2–4.3)	5.42 (7.2–4.4)

**Table 3:** Therapeutic responses of patients treated with Mb and chloroquine syrups from day 3 to day 28.

Parameters	Group I: Mb (*n* = 21)	Group II: CQ (*n* = 21)
Body temperature °C, mean		
Day 3	36.75 (38.0–36.2)	36.55 (37–36)
Day 7	36.8 (38.1–36.2)	36.87 (37.7–36.5)
Day 14	36.69 (37.5–35.9)	36.6 (37.7–35.7)
Day 28	36.58 (37.4–35.1)	36.82 (37.5–36)
Geometric mean parasitaemia/mm^3^ ± SD		
Day 3	823,24 ± 778	771,81 ± 584
Day 7	480,00 ± 451,69	449,14 ± 448,43
Day 14	359,62 ± 377,64	432,00 ± 428,34
Day 28	153,14 ± 250,20	165,71 ± 311,87
Blood glucose (mmol/l)		
Day 7	5.5 (7.3–4.5)	5.38 (7.1–4.1)
Day 14	5.53 (6.9–4.3)	5.63 (9–4.6)
Day 28	5.34 (7.2–4.6)	5.64 (7.8–4.2)
Hematocrit (%)		
Day 7	34.76 (40–29)	33.67 (40–28)
Day 14	29.81 (36–20)	28.95 (36–24)
Day 28	31.05 (40–26)	31.81 (36–26)
Haemoglobin concentration, mean g/dl		
Day 7	7.78 (10–6)	8.17 (11–6.2)
Day 14	8.95 (11.1–6)	8.91 (11–7.1)
Day 28	8.95 (10.8–6.8)	9.18 (12–7)
